# Effects of High-Fat and High-Fat/High-Sucrose Diet-Induced Obesity on PVAT Modulation of Vascular Function in Male and Female Mice 

**DOI:** 10.3389/fphar.2021.720224

**Published:** 2021-09-10

**Authors:** Jamaira A. Victorio, Daniele M. Guizoni, Israelle N. Freitas, Thiago R. Araujo, Ana P. Davel

**Affiliations:** ^1^ Department of Structural and Functional Biology, Laboratory of Vascular Biology, Institute of Biology, University of Campinas, Campinas, Brazil; ^2^ Department of Structural and Functional Biology, Obesity and Comorbidities Research Center-OCRC, Institute of Biology, University of Campinas, Campinas, Brazil

**Keywords:** obesity, perivascular adipose tissue, sex differences, resistance arteries, endothelial dysfunction

## Abstract

Increased adiposity in perivascular adipose tissue (PVAT) has been related to vascular dysfunction. High-fat (HF) diet-induced obesity models are often used to analyze the translational impact of obesity, but differences in sex and Western diet type complicate comparisons between studies. The role of PVAT was investigated in small mesenteric arteries (SMAs) of male and female mice fed a HF or a HF plus high-sucrose (HF + HS) diet for 3 or 5 months and compared them to age/sex-matched mice fed a chow diet. Vascular responses of SMAs without (PVAT-) or with PVAT (PVAT+) were evaluated. HF and HF + HS diets increased body weight, adiposity, and fasting glucose and insulin levels without affecting blood pressure and circulating adiponectin levels in both sexes. HF or HF + HS diet impaired PVAT anticontractile effects in SMAs from females but not males. PVAT-mediated endothelial dysfunction in SMAs from female mice after 3 months of a HF + HS diet, whereas in males, this effect was observed only after 5 months of HF + HS diet. However, PVAT did not impact acetylcholine-induced relaxation in SMAs from both sexes fed HF diet. The findings suggest that the addition of sucrose to a HF diet accelerates PVAT dysfunction in both sexes. PVAT dysfunction in response to both diets was observed early in females compared to age-matched males suggesting a susceptibility of the female sex to PVAT-mediated vascular complications in the setting of obesity. The data illustrate the importance of the duration and composition of obesogenic diets for investigating sex-specific treatments and pharmacological targets for obesity-induced vascular complications.

## Introduction

Excessive body fat storage (i.e., increased intake energy with reduced energy expenditure) is a major feature of obesity. This condition results in chronic low-grade inflammation presenting a more complex resolution and represents a risk factor for the development and/or worsening of several chronic diseases, including cardiovascular diseases (CVDs) (Caballero et al., 2008; World Health Organization, 2019). Over time (1975–2016), the prevalence of obesity has increased in both sexes; however, women have shown a higher prevalence of obesity than men (Abarca-Gómez et al., 2017). Conversely, men have a higher prevalence of increased blood pressure than women (Zhou et al., 2017). Despite the knowledge of sex-specific differences in the development of obesity and CVDs, few studies have covered obesity-related vascular (dys)function in males and females.

Adipose tissue is recognized as a secretory organ, and its anatomical location may predict modulation of adjacent tissues/organs during the development of obesity (Lim and Meigs, 2014; Vishvanath and Gupta, 2019). Perivascular adipose tissue (PVAT), a fat depot evolving almost all vessels, modulates vascular function, and thus, changes in morphology and vasoactive factors synthesized or secreted by PVAT may result in altered vascular function. Studies have demonstrated that obesity in humans is correlated with an increase in the volume of PVAT (Schlett et al., 2009; Wagner et al., 2012) and results in loss of the PVAT anticontractile effect (Greenstein et al., 2009). Using experimental models of male obesity, several studies have reported an impaired anticontractile effect of PVAT (da Costa et al., 2017; Gil-Ortega et al., 2014; Han et al., 2018; Ma et al., 2010; Withers et al., 2017; Xia et al., 2016; Xu et al., 2012), although an increased PVAT anticontractile effect has also been demonstrated in response to high-calorie diets (Gil-Ortega et al., 2010; Dos Reis Costa et al., 2021). In addition to the controversial data in male obesity experimental models, much less is known about PVAT function in females, especially in the setting of obesity (Victorio et al., 2020). Sexual dimorphism in PVAT immune cell content in response to a high-fat (HF) diet was recently demonstrated (Kumar et al., 2021), but it is still unclear whether obesogenic diets differentially impact PVAT anticontractile function in males and females over time.

Animal models are key tools for understanding and treating obesity comorbidities (Barrett et al., 2016). A HF diet with 45% or 60% of calories from fat is the most commonly used obesogenic diet in rodents. Although 60% fat diets induce a faster and more obese model, a 45% fat diet seems to be more relevant for human physiology (Speakman, 2019). In addition, a diet with 60% fat has less sucrose than a diet with 45% fat, and differences in total sugar and sugar sources may influence cardiovascular outcomes (Xi et al., 2015; Narain et al., 2016). Comparisons between sexes suggest that Western diets (high-fat and high-sucrose diets) eliminate the protective effect of female sex on endothelial function (Hunter et al., 2017; Davel et al., 2018b), but the impact on PVAT control of vascular tone is still unknown. The present study investigated whether two different HF obesogenic diets (60% HF versus 45% HF plus high sucrose) impact PVAT anticontractile function differentially in males and females over time.

## Methods

### Animal Model

Male and female C57Bl6/J mice were purchased from the Multidisciplinary Center for Biological Investigation on Laboratory Animal Science of the University of Campinas. Animals were maintained in a room with controlled humidity and temperature (22 ± 2°C) under a 12 h:12 h light/dark cycle and received water and a chow diet (CD) ad libitum. At 2 months of age, the mice were divided to receive CD (3.86 kcal/g), a very HF (60% calories from fat; 5.55 kcal/g) diet, or a HF plus high-sucrose (HF + HS, 45% calories from fat and 30% from sucrose; 4.85 kcal/g) diet purchased from Quimtia^©^ (Colombo, PR, Brazil) and PragSoluções^©^ (Jaú, SP, Brazil), respectively, for a period of 3 or 5 months. Nutritional information for each diet is available in the supplementary material (Supplementary Table S1). Measurement of food consumption was performed weekly during the feeding protocol.

This study was approved by the Ethics Committee on Animal Use of the University of Campinas (protocol no. 4914-1/2018, 5474-1/2020) and carried out in accordance with the National Board of Animal Experimentation Control (CONCEA).

### Blood Pressure Measurement

Systolic blood pressure (SBP) was assessed via tail-cuff plethysmography (LE5001 Pressure meter, Panlab, Harvard Apparatus, Barcelona, Spain) on the day before tissue collection and vascular function was assessed. Animals were restrained for 2 min before the first SBP measurement, which was considered successful when the mouse did not move, and a clear pulse was observed. Ten sequential measurements from each animal were registered. The SBP is expressed as the average of the ten measurements.

### Body Parameters, Tissue Collection, and Biochemical Profile

Final body weight was obtained at each period. The day before tissue collection and vascular study performance and after SBP measurement, fasting glucose was measured in 4 h fasted mice by cutting the tail tip and using OneTouch Ultra^®^ and reactive strips. At the end of each time point in the diet protocol, animals were anesthetized with a superdose of anesthesia (ketamine, 240 mg/kg; xylazine, 30 mg/kg). On the day we conducted the vascular study, blood samples were collected from fed animals by cardiac puncture and centrifuged to obtain serum samples, which were stored at −80°C until analyzing insulin and adiponectin levels. The peritoneal cavity was opened, and the mesenteric bed, perigonadal adipose tissue, and sex organs (testes/uterus) were removed. The fat pads and sex organs were weighed. The mesenteric bed was placed in ice-cold Krebs–Henseleit solution (KHS; in mM: NaCl 115; KCl 4.6; CaCl2·2H2O 2.5; KH2PO4 1.2; MgSO4·7H2O 12.4; NaHCO3 25; glucose 5.5) for vascular study.

### Vascular Study

The second- and third-order mesenteric arteries (artery diameter ∼130 µm) were dissected in ice-cold KHS. In some segments, adjacent PVAT was left intact (PVAT+), whereas, in others, PVAT was dissected out (PVAT-). Arteries were cut into 1–2 mm rings, mounted on a 20 µm diameter wire in a multichannel myograph (Model 610M, DMT A/S, Aarhus NA, Denmark), and left to equilibrate for at least 20 min before normalization using a standardized procedure (Mulvany and Halpern, 1977; Wenceslau et al., 2021). A normalization protocol that considers the inner circumference and wall tension was performed (Saxton et al., 2018). After normalization, the arteries were left for an additional 20 min equilibration period at 37°C and bubbled at 95% O_2_/5% CO_2_ to maintain a pH of 7.4. Next, arteries were contracted with 60 mM KCl twice, first for 3 min following 15 min of equilibration and a second time for 10 min, to establish the maximum contractile response to KCl and confirm that the removal of PVAT did not damage the vascular smooth muscle. The maximum KCl response was similar in arteries with or without PVAT in males and females (Supplementary Figure S1). After a 30 min equilibration period, PVAT+ and PVAT- arteries were precontracted (50% of maximum KCl contraction) with phenylephrine (1 µM for PVAT- arteries and 10 µM for PVAT+ arteries); then, concentration-response curves to acetylcholine (0.1 nM–30 µM) were obtained to evaluate endothelium-dependent relaxation. To evaluate the anticontractile effect of PVAT, arteries were contracted with phenylephrine (1 nM–0.1 mM).

## Materials

Acetylcholine and phenylephrine were obtained from Sigma-Aldrich (Merck KGaA, Darmstadt, Germany). Salts for KHS were obtained from Labsynth (Diadema, Brazil). A mouse insulin ELISA was obtained from Merck (Merck KGaA; cat. #EZRMI-13K). A mouse adiponectin/Acrp30 DuoSet ELISA (DY1119) was obtained from R&D Systems (Minneapolis, United States).

### Statistical Analysis

The results are expressed as the mean ± SEM and were analyzed via one-way or two-way ANOVA with a Bonferroni multiple comparison test for data that satisfied the Shapiro–Wilk normality test. When data did not pass the normality test, Mann–Whitney or Kruskal–Wallis tests were applied. *p* values <0.05 were considered to indicate a significant difference. The contractile response to phenylephrine is expressed in mN/mm. Relaxation responses are expressed as a percentage (%) of the precontraction with phenylephrine. The maximum response (Rmax) and potency (the negative logarithm to base 10 of the molar concentration of an agonist producing 50% of the maximal effect; pEC_50_) were calculated in each concentration-response curve. In figures, PVAT- data are represented by white symbols and PVAT+ data by blue shapes for males and pink for females. Statistical analysis was performed with GraphPad Prism 8.4.3 software (GraphPad Software, San Diego, CA, United States).

## Results

### HF and HF + HS Obesity Models in Males and Females

In both sexes, energy intake was similar between the HF and CD groups, while food consumption was lower in the HF group than that in the CD group (Supplementary Table S2). In contrast, compared to the CD group, males and females fed a HF + HS diet exhibited increased energy intake, while food consumption remained reduced in males and similar in females (Supplementary Table S2). In male and female mice fed with HF + HS diet, food consumption was higher than that in mice fed with HF diet (Supplementary Table S2), suggesting that adding sugar to a HF diet could result in a more palatable diet.

Feeding of a HF or HF + HS diet for 3 or 5 months increased body weight and perigonadal fat adiposity in males and females compared to those mice in the respective sex-matched CD group (Supplementary Table S2). The HF and HF + HS diets increased fasting glucose in both sexes at 3 and 5 months. The HF + HS diet induced a time-dependent increase in fasting glucose in males and females and in adiposity in females (Supplementary Table S2). Five months of HF diet feeding resulted in a significant threefold increase in insulin levels, and although the insulin levels doubled in the HF + HS group compared to the CD group, the difference did not reach significant values (male: CD = 0.92 ± 0.1; HF = 3.05 ± 0.2*; HF + HS = 1.96 ± 0.6 ng/ml; *n* = 3–4/group; *p < 0.05 vs. CD). Female obese mice showed doubled plasma insulin levels after 5 months of a HF or HF + HS diet compared to female CD-fed mice (female: CD = 0.57 ± 0.08; HF = 1.18 ± 0.01*; HF + HS = 1.23 ± 0.19* ng/ml; n = 3–4/group; *p < 0.05 vs. CD). Adiponectin levels were not changed by the diet protocol (male: CD = 366 ± 164; HF = 266 ± 69; HF + HS = 210 ± 23 pg/ml; female: CD = 212 ± 23; HF = 228 ± 31; HF + HS = 300 ± 90 pg/ml; *n* = 4/group; *p* > 0.05). SBP and testis and uterine weight did not differ between groups (Supplementary Table S2).

### Anticontractile PVAT Function was Preserved in Males but Impaired in Females Following HF or HF + HS Diet Feeding

**FIGURE 1:**
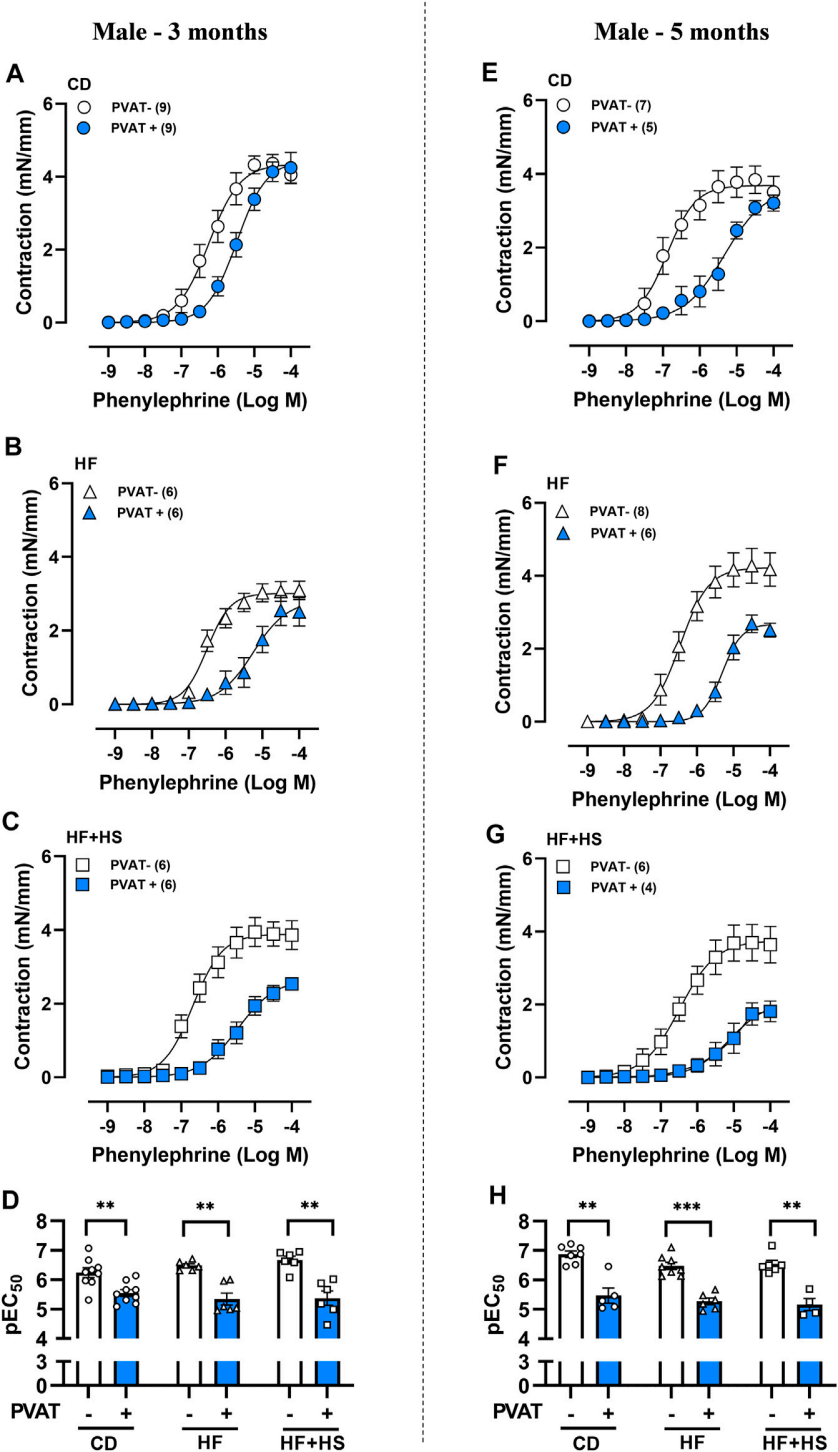
Obesogenic diets did not impair the anticontractile effect of mesenteric PVAT in male mice. Concentration-response curves to phenylephrine in mesenteric arteries without PVAT (PVAT-; white symbols) or in the presence of adjacent PVAT (PVAT +; blue filled symbols) of male mice fed a chow diet (CD; **A,E;** circle symbols), high-fat diet (HF; **B,F;** triangle symbols) or HF plus high-sucrose diet (HF + HS; **C,G;** square symbols) for 3 (left panel) or 5 (right panel) months. Bar graphs show the potency of the response to phenylephrine (pEC_50_) **(D,H)** in PVAT- (white bars) and PVAT+ (blue filled bars) arteries. The experimental number used is in parenthesis. **p < 0.01; ***p < 0.001 vs. PVAT- arteries (Mann–Whitney *U* test).

**FIGURE 2:**
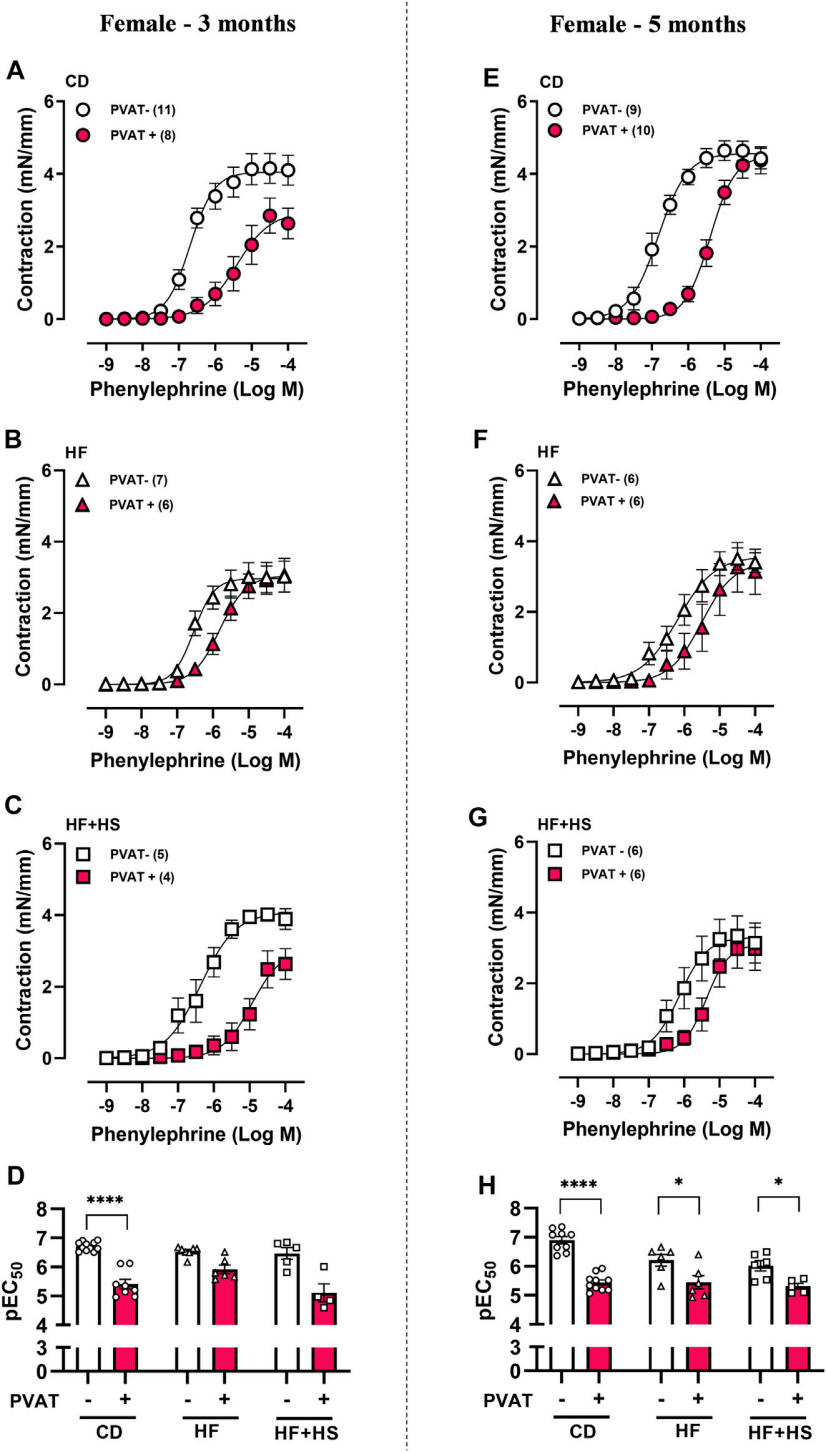
The anticontractile effect of mesenteric PVAT is reduced by high-fat and high-fat plus high-sucrose diets in female mice. Concentration-response curves to phenylephrine in mesenteric arteries without PVAT (PVAT-; white symbols) or in the presence of adjacent PVAT (PVAT +; pink filled symbols) from female mice fed a chow diet (CD; **A,E;** circle symbols), high-fat diet (HF; **B,F;** triangle symbols), or HF plus high-sucrose diet (HF + HS; **C,G;** square symbols) for 3 (left panel) or 5 (right panel) months. Bar graphs show the potency of the response to phenylephrine (pEC_50_) **(D,H)** in PVAT- (white bars) and PVAT+ (pink filled bars) arteries. The experimental number used is in parenthesis. ****p < 0.0001 vs. PVAT- (pEC_50_ 3 months) (Mann–Whitney *U* test). *p < 0.05, ****p < 0.0001 vs. PVAT- (pEC_50_ 5 months) (two-way ANOVA).

The anticontractile effect of PVAT was analyzed by comparing the contraction induced by phenylephrine in small mesenteric arteries with and without adjacent PVAT. In males fed a HF or HF + HS diet for 3 months, the anticontractile effect of PVAT was similar to that in CD sex-matched mice, as demonstrated by a similar LogEC_50_ (pEC_50_) and a reduced Rmax to phenylephrine in PVAT + arteries from the HF + HS groups compared to those from the CD group (Figures 1A–D; Supplementary Table S3). When male mice were fed a HF or HF + HS diets for 5 months, the anticontractile effect of PVAT was greater than that observed in CD mice (Figures 1E–H; Supplementary Table S3). In contrast, in females, the anticontractile effect of PVAT was impaired by both obesogenic diets, evidenced by a reduction in pEC_50_ to phenylephrine in PVAT + arteries of HF and HF + HS diet mice compared to the matched CD group at 3 and 5 months (Figures 2A–H). Rmax did not differ between female groups (Supplementary Table S3).

### The HF + HS Diet Resulted in PVAT-Mediated Endothelial Dysfunction Faster in Females than in Males

**FIGURE 3:**
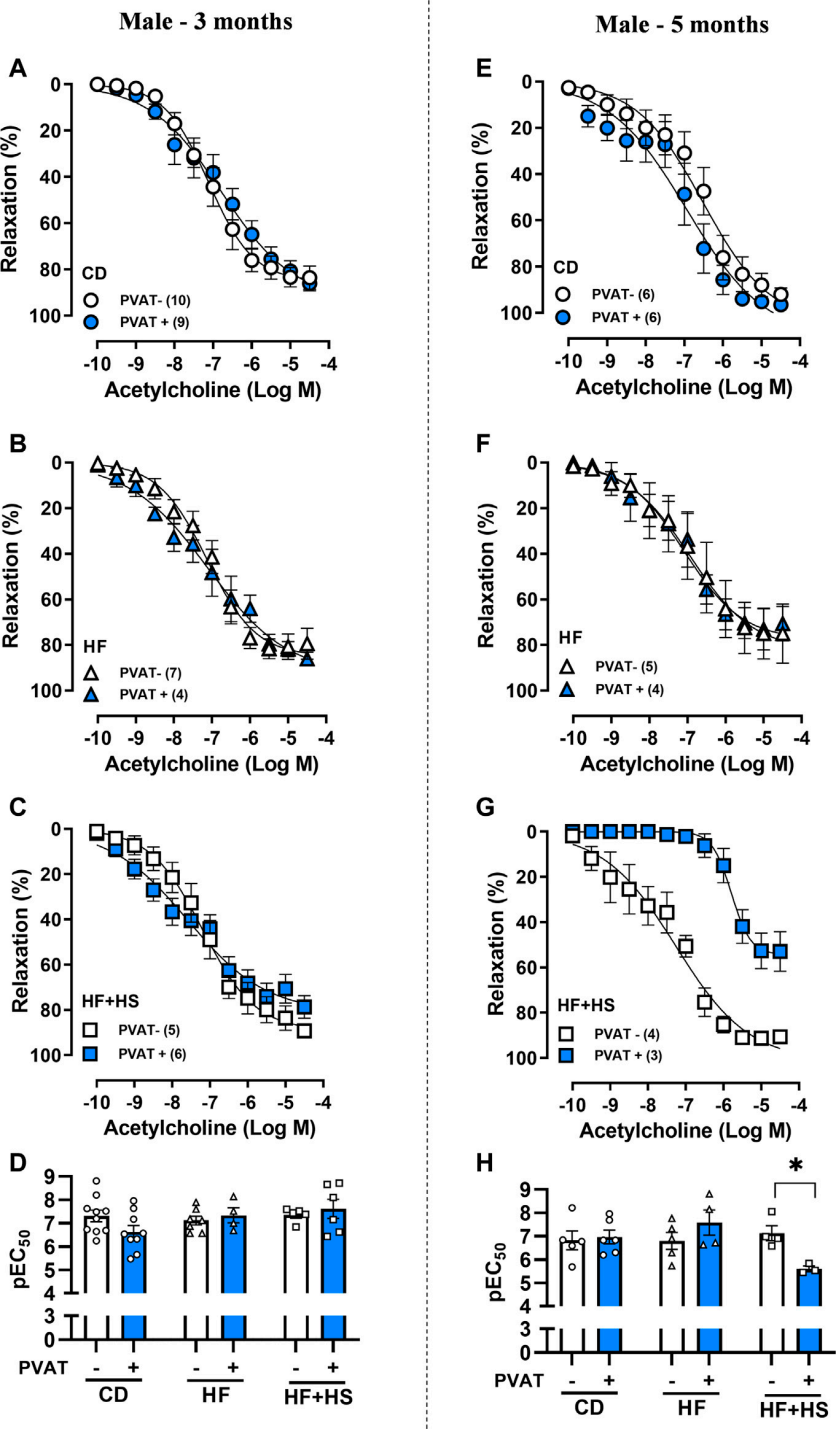
Effect of adjacent PVAT on acetylcholine-induced relaxation of mesenteric arteries from male mice. Concentration-response curves to acetylcholine in mesenteric arteries without PVAT (PVAT-; white symbols) or in the presence of adjacent PVAT (PVAT +; blue filled symbols) from male mice fed a chow diet (CD; **A**,**E;** circle symbols), high-fat diet (HF; **B**,**F;** triangle symbols) and HF plus high-sucrose diet (HF + HS; **C**,**G;** square symbols) for 3 (left panel) or 5 (right panel) months. Bar graphs show the potency of the response to acetylcholine (pEC_50_) **(D,H)** in PVAT- (white bars) and PVAT+ (blue filled bars) arteries. The experimental number used is in parenthesis. *p < 0.05 (two-way ANOVA).

Next, we evaluated PVAT modulation of acetylcholine-induced endothelium-dependent relaxation. The presence of PVAT did not impact the acetylcholine response in mesenteric arteries from CD or HF diet male mice (Figures 3A–H). Otherwise, following 5 months of a HF + HS diet, PVAT+ arteries exhibited impaired acetylcholine-induced relaxation compared to PVAT- arteries (Figure 3G), as demonstrated by the reduced pEC_50_ (Figure 3H) and Rmax to acetylcholine in PVAT+ arteries (Rmax in 5-month HF + HS males: PVAT- = 93.5 ± 1.5 vs. PVAT+ = 56.5 ± 7.1%, *n* = 3–4; *p* < 0.05).

**FIGURE 4:**
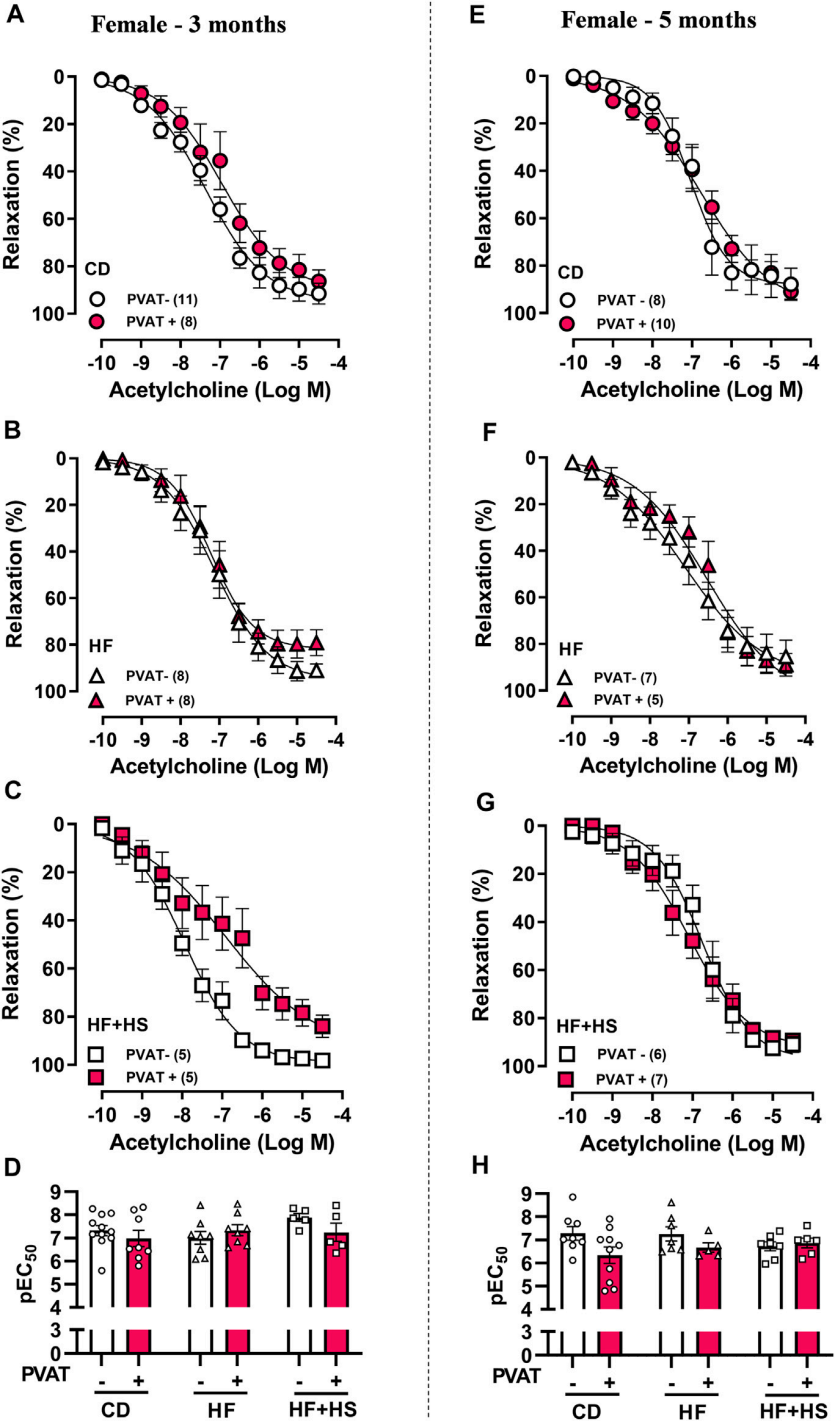
Effect of adjacent PVAT on acetylcholine-induced relaxation of mesenteric arteries from female mice. Concentration-response curves to acetylcholine in mesenteric arteries without PVAT (PVAT-; white symbols) or in the presence of adjacent PVAT (PVAT +; pink filled symbols) from female mice fed a chow diet (CD; **A**,**D;** circle symbols), high-fat diet (HF; **B**,**E;** triangle symbols), and HF plus high-sucrose diet (HF + HS; **C**,**F**; square symbols) for 3 (left panel) or 5 (right panel) months. Bar graphs show the potency of the response to acetylcholine (pEC_50_) **(D**,**H)** in PVAT- (white bars) and PVAT+ (pink filled bars). The experimental number used is in parenthesis. *p* > 0.05 (two-way ANOVA).

Similar to males, the presence of PVAT did not impact the acetylcholine response in mesenteric arteries from the CD or HF diet groups (Figures 4A–H). Nevertheless, the HF + HS diet impacted acetylcholine-induced relaxation earlier in females (Figures 4A–C); after 3 months of a HF + HS diet, PVAT+ arteries exhibited a reduced Rmax to acetylcholine compared to PVAT- arteries (Rmax in 3-month HF + HS females: PVAT- = 97.4 ± 0.6 vs. PVAT+ = 78.3 ± 5.3%, *n* = 5; *p* < 0.05) with no changes in pEC_50_ (Figure 4D). When the HF + HS diet was prolonged for up to 5 months, PVAT+ arteries presented the same acetylcholine response as PVAT- arteries with no changes in pEC_50_ or Rmax (Figures 4G,H). Notably, small mesenteric arteries in the female 5-month HF + HS diet group exhibited endothelial dysfunction independent of the presence of PVAT, since in this group, the acetylcholine-induced relaxation curve was shifted to the right in PVAT- arteries (pEC_50_ PVAT-: 3 months = 7.8 ± 0.1 vs. 5 months = 6.8 ± 0.2, *n* = 5–6; *p* < 0.05).

## Discussion

In this study, we evaluated the prolonged effects of two obesogenic HF diets (HF and HF + HS diets) on small mesenteric PVAT function in male and female mice. In males, 3 and 5 months of obesogenic diet feeding resulted in similar anticontractile effects of PVAT. In contrast to males, in females, we found that prolonged HF or HF + HS diet feeding resulted in an impaired anticontractile effect of PVAT. The HF + HS diet resulted in PVAT-mediated endothelial dysfunction as early as 3 months after obesogenic feeding in females but only after 5 months in males. The presence of PVAT did not impact acetylcholine-induced endothelial relaxation in mice fed a HF diet alone, suggesting that the addition of sucrose accelerates PVAT dysfunction in response to obesogenic diets. PVAT dysfunction in response to the HF and HF + HS diets was observed early in females compared to age-matched males, suggesting a susceptibility of the female sex to PVAT-mediated vascular complications in the setting of obesity. The data highlight the importance of an obesogenic diet and the duration of the feeding protocol for investigating treatments and pharmacological targets for obesity-induced vascular complications in males and females.

The anticontractile effect of PVAT was first described in rat thoracic aorta (Soltis and Cassis, 1991), followed by other vascular beds, including small mesenteric arteries (Aoqui et al., 2014; Gil-Ortega et al., 2014; Withers et al., 2017). It has been suggested that the presence of cardiometabolic risk factors, such as obesity, alters the secretory pattern of PVAT and might result in vascular dysfunction (Victorio and Davel, 2019). PVAT-dependent vascular dysfunction has been demonstrated in the aorta (da Costa et al., 2017; Ketonen et al., 2010) and mesenteric resistance arteries (Agabiti-Rosei et al., 2014; Gil-Ortega et al., 2014; Withers et al., 2017) in rat and mouse obese models. Several mechanisms have been described to be involved in PVAT dysfunction associated with obesity, which seems to depend on PVAT location and the duration of obesogenic diets and local/circulating adipokines (Victorio and Davel, 2019; Victorio et al., 2020). Adiponectin was demonstrated to act as a PVAT-derived anticontractile factor and could be a promising mediator of PVAT function in female mice. Here, we observed similar plasma adiponectin levels between the obese and control groups, independent of sex. Plasma adiponectin in male obesity has been demonstrated to be increased (Saxton et al., 2021), unchanged (Sousa et al., 2019), or reduced (Han et al., 2018). Adiponectin levels can differ with the time of induced obesity, with increased levels in early phases and reduced levels in prolonged phases (Victorio and Davel, 2019). Moreover, plasma adiponectin cannot represent what happens in local modulation of vascular function by PVAT, as demonstrated by Saxton and collaborators who have observed increased plasma adiponectin in obesity but reduced adiponectin content in PVAT (Saxton et al., 2021). Therefore, more studies are needed to evaluate the effects of different diets on PVAT adipokines, especially in females in the setting of obesity.

Time-dependent resistance of PVAT to dysfunction was observed in male obese mice. An increased anticontractile effect of PVAT associated with adaptative nitric oxide (NO) overproduction was found in the mesenteric arteries from male mice fed a high-fat diet (45% fat) for 8 weeks (Gil-Ortega et al., 2010) and in the aortas from mice fed a carbohydrate-enriched diet for 4 weeks (Dos Reis Costa et al., 2021). However, after 32 weeks of a 45% fat diet, altered redox status resulted in PVAT dysfunction in mesenteric arteries (Gil-Ortega et al., 2010; Gil-Ortega et al., 2014). In the present study, 3 or 5 months of a very HF diet (60% fat) did not impair either the anticontractile effect of PVAT in response to phenylephrine or the endothelium-dependent relaxation in response to acetylcholine. PVAT-dependent endothelial dysfunction was observed only after 5 months of the HF + HS diet in males. This suggests that the association of a HF and HS diet (i.e., Western diet) may accelerate PVAT and vascular dysfunction compared to a HF diet.

Since discovering the anticontractile effect of PVAT, it was expected that PVAT could increase acetylcholine-induced relaxation, but in our study, we did not observe an effect of PVAT in response to acetylcholine in male and female control mice. Similar to what was observed here, several studies have demonstrated that the presence of PVAT does not affect acetylcholine-induced relaxation in the aorta (Victorio et al., 2016; Hou et al., 2017; Simplicio et al., 2017; Sousa et al., 2019) or mesenteric arteries (Briones et al., 2012; Kagota et al., 2017). However, we cannot exclude the possibility that PVAT can act as a mechanical obstacle or diffusion limitation to agonists (Li et al., 2013; Mendizabal et al., 2013). In the setting of obesity, previous studies have demonstrated that PVAT-mediated impaired acetylcholine-induced relaxation in mice fed HF diet seems to be endothelium-dependent since relaxation to sodium nitroprusside was not altered (Sousa et al., 2019; Sousa et al., 2021). Both NO and endothelium-dependent hyperpolarization factor (EDH) seem to contribute to the endothelium-dependent relaxation in small mesenteric arteries from male and female mice (Hwa et al., 1994; Davel et al., 2018b). Nevertheless, NO has a greater contribution than EDH in males, while EDH contributes more than NO to endothelial relaxation induced by acetylcholine in females (Davel et al., 2018b). In the setting of HF-induced obesity, there is a reduction in endothelial NO contribution, which compensates for increased EDH participation; however, in females, an impaired EDH participation results in impaired acetylcholine-induced relaxation (Davel et al., 2018b). Previous studies using HF or cafeteria diet have suggested that impaired aortic PVAT-derived NO is associated with a reduction in acetylcholine-induced relaxation in male mice (Xia et al., 2016; Lang et al., 2019). Further studies are needed to investigate the relative contribution of NO/EDH in the PVAT-mediated endothelial dysfunction in resistance arteries of males and females in response not only to obesity but also to other cardiovascular risk factors.

Reduced Rmax to phenylephrine in arteries with adjacent PVAT was observed following prolonged HF or HF + HS diet feeding in males. Silva and collaborators (Silva et al., 2016) have previously demonstrated that a high sugar diet, in addition to inducing endothelial dysfunction, upregulates the TNFα and iNOS pathways, decreasing vascular contractility in the aortas of obese male animals. Therefore, we cannot exclude a proinflammatory effect of a prolonged HF + HS diet on PVAT function. Lang and collaborators have demonstrated that a Western diet containing high sugar and dense foods accessible in Western societies impacted the aortic PVAT function more profoundly than a HF diet (45% fat) (Lang et al., 2019). Although a cafeteria diet has been demonstrated to be similar to a human diet and induced obesity, it can change feeding behavior (Martire et al., 2015); thus, it may be difficult to compare results in the literature because there is no standard protocol (Lang et al., 2019). Here, we used a chow diet as a standard diet, but a nonfat diet has been proposed for use as a control for fat-enriched diets (Lang et al., 2019), which may represent a limitation. However, as we meant to compare the effect of PVAT function after HF diet consumption to that a healthy/lean situation, we believe that the chow diet represented a better control.

Sugar consumption has been correlated with adverse effects on HDL and triglyceride levels, which can accelerate atherosclerosis (Howard and Wylie-Rosett, 2002). In addition, high sugar consumption may worsen diabetes control, and the combination of sugar with high fat promotes AGEs formation, which is involved in vascular complications associated with diabetes (Yonekura et al., 2005; Delbin et al., 2012). In women, a diet high in refined carbohydrates was associated with an increased risk of developing coronary heart disease (Liu et al., 2000). These previous studies have reinforced the importance of investigating the influence of high-calorie diets associated with high sugar content on the mechanisms that modulate vascular and PVAT function, especially in females.

In comparison to males, far less is known about functional PVAT changes in vascular tone in the setting of female obesity, which was a focus of the current study. A recent study revealed that sex-specific changes in the number of immune cells in mesenteric PVAT occurring with a HF diet (60% fat) became more prominent with the development and progression of obesity (Kumar et al., 2021). Here, we found that prolonged obesogenic diet feeding differentially impacted PVAT regulation of vascular responses in mesenteric arteries in males and females. HF and HF + HS diet feeding impaired the anticontractile effect of PVAT in females but not in males. In addition, 3 months of a HF + HS diet resulted in PVAT-mediated endothelial dysfunction in females, which was observed in males after 5 months of the same diet. Therefore, our data suggest that mesenteric PVAT in females is more susceptible to obesity-induced dysfunction than that in males. This is consistent with previous studies demonstrating that the female sex is more affected by endothelial dysfunction and vascular stiffness caused by obesity and type-2 diabetes (DeMarco et al., 2015; Jia et al., 2016; Davel et al., 2018a). Although we did not evaluate the levels of sex hormones, gonad weights (uterus and tests) were not significantly altered by either the HF or HF + HS diet, consistent with previous studies (Bruder-Nascimento et al., 2017; Faulkner et al., 2021). Loss of female sex hormones was not reported as a mechanistic switch for cardiovascular complications in obese female mice (Barris et al., 2021). A limitation of our study is that we did not investigate the mechanisms or anatomical differences underlying the sex differences in PVAT-mediated vascular complications in obesity, which need to be addressed in future investigations.

## Conclusion

The results suggest that prolonged obesogenic diets impact mesenteric PVAT function more profoundly in females than in males, as after 3 and 5 months of HF or HF + HS feeding, impairment in the anticontractile PVAT effect was only observed in females. Our data also revealed that the combination of a HF diet with high sucrose accelerated PVAT dysfunction compared to a HF diet alone, resulting in PVAT-mediated endothelial dysfunction in males and females. PVAT-mediated endothelial dysfunction with a HF + HS diet occurs earlier in females than in males. Taken together, the data suggest a susceptibility of the female sex to PVAT-mediated vascular complications in the setting of obesity and highlight the importance of an obesogenic diet and the duration of the feeding protocol for investigating treatments and pharmacological targets for obesity-induced vascular complications in males and females.

## Data Availability

The original contributions presented in the study are included in the article/Supplementary Material; further inquiries can be directed to the corresponding authors.
