# Supplementary material for: Effects of High-Fat and High-Fat/High-Sucrose Diet-Induced Obesity on PVAT Modulation of Vascular Function in Male and Female Mice
Source: Front Pharmacol. 2021 Sep 10;12:720224. doi: 10.3389/fphar.2021.720224 (PMC8460896; doi:10.3389/fphar.2021.720224)
Supplement: Supplementary file 1 [file DataSheet1.PDF]

Supplemental Material

Table S1. Nutritional information of the chow (CD), high-fat (HF), and high-fat with sucrose (HF+HS) diets.

**Diets**		**CD***		**HF****		**HF+HS****	
		**%**	**cal/g**	**%**	**cal/g**	**%**	**cal/g**
**Fat**	Energy	11.9	360	61.6	3420	44.5	2160
**Protein**	Energy	23.6	880	14.6	812	14.7	712
**Carbohydrate**	Energy	62.2	2480	23.7	1318	40.8	1978
	Sucrose	-	-	15	197.3	30	593.4
**Vitamins**	Energy	2.0	49.6	1	13.15	1	19.78
**Energy (kcal/g)**		3.86		5.55		4.85	

* Nuvilab CR-1©, nutritional information provided by Quimtia©, Brazil.

** Nutritional information provided by PragSoluções©, Brazil.

Table S2. Body parameters from male and female mice fed with chow diet (CD), high-fat (HF) and high-fat with high-sucrose (HF+HS) diet.

Groups	Male						Female					
	3 months			5 months			3 months			5 months		
	**CD**	**HF**	**HF+HS**	**CD**	**HF**	**HF+HS**	**CD**	**HF**	**HF+HS**	**CD**	**HF**	**HF+HS**
**Body weight (g)**	26.9±0.4 (8)	46.8±0.5 (7) *	44.1±1.6 (6) *	27.9±0.8 (7)	51.4±1.5 (6) *	46.6±1.4 (8) *$^{\#}$	22.5±0.4 (10)	35.1±1.3 (8) *	32.0±2.7 (5) *	22.8±0.3 (8)	44.8±2.2 (6) *$^{\$}$	37.5±1.2 (6) *$^{\#}$
**Energy intake (kcal/day/animal)**	15.5±1.3 (8)	15.1±1.7 (7)	17.3±1.6*$^{\#}$ (6)	15.7±0.4 (7)	15.1±0.4 (6)	17.5±0.4*$^{\#}$ (8)	15.1±0.5 (10)	17.4±0.5 (8)	18.4±1.07* (5)	15.3±0.3 (8)	16.8±0.4 (6)	18.8±0.6*$^{\#}$ (6)
**Food consumption (kcal/day/animal)**	4.5±0.1 (8)	2.7±0.1* (7)	3.4±0.09*$^{\#}$ (6)	4.5±0.1 (7)	2.7±0.06* (6)	3.4±0.07*$^{\#}$ (8)	3.9±0.1 (10)	2.9±0.09$^{\&}$ (8)	3.7±0.2§ (5)	3.9±0.08 (8)	2.9±0.06$^{\&}$ (6)	3.8±0.1§ (6)
**Perigonadal fat weight (mg/cm)**	223.7±41 (8)	1192.0±376 (7) *	1280.9±231 (5) *	331.1±49 (8)	919.1±61 (6) $^{\&}$	936.3±125 (5) $^{\&}$	114.1 ± 18 (10)	475.8±115 (6) *	1075.2 ± 220 (5) *$^{\#}$	209.3±15 (8)	1851.9±189 (5) *$^{\$}$	1553.3±111 (7) *$^{\$}$
**Fasting glucose (mg/dL)**	131±7 (7)	237±12 (7) *	172±4 (6) *$^{\#}$	146±4 (8)	203±7 (6) *$^{\$}$	222±18 (4) *$^{\$}$	103±3 (6)	165±8 (8) *	133±2 (5) *$^{\#}$	112±4 (8)	143±8 (6) *	179±9 (4) *$^{\#\$}$
**SBP (mmHg)**	115±2 (8)	117±2 (7)	107±5 (6)	107±10 (7)	117±15 (6)	103±7 (5)	117±2 (10)	122±5 (8)	110±4 (5)	113±4 (8)	108±10 (6)	116±4 (6)
**Gonadal weight (mg/cm)**	118.1±2 (10)	119.2±3 (7)	118.4±3 (5)	124.1±3 (8)	126.6±5 (6)	1225±4 (5)	61.6±5 (10)	49.8±4 (5)	73.3±9 (5)	53.5±4 (8)	76.0±4 (4)	59.4±6 (7)

CD: chow diet; HF: high fat; HF+HS: high fat plus sucrose; SBP: systolic blood pressure. One-way ANOVA with Tukey's post hoc*$p<0.05$ *vs.* CD; $^{\#}$ $p<0.05$ *vs.* HF. Two-way ANOVA with Bonferronis' test. $^{\$}p<0.05$ vs. the same parameter in 3 mo. Kruskal-Wallis test $^{\&}p<0.05$ *vs.* CD; § $p<0.05$ *vs.* HF.

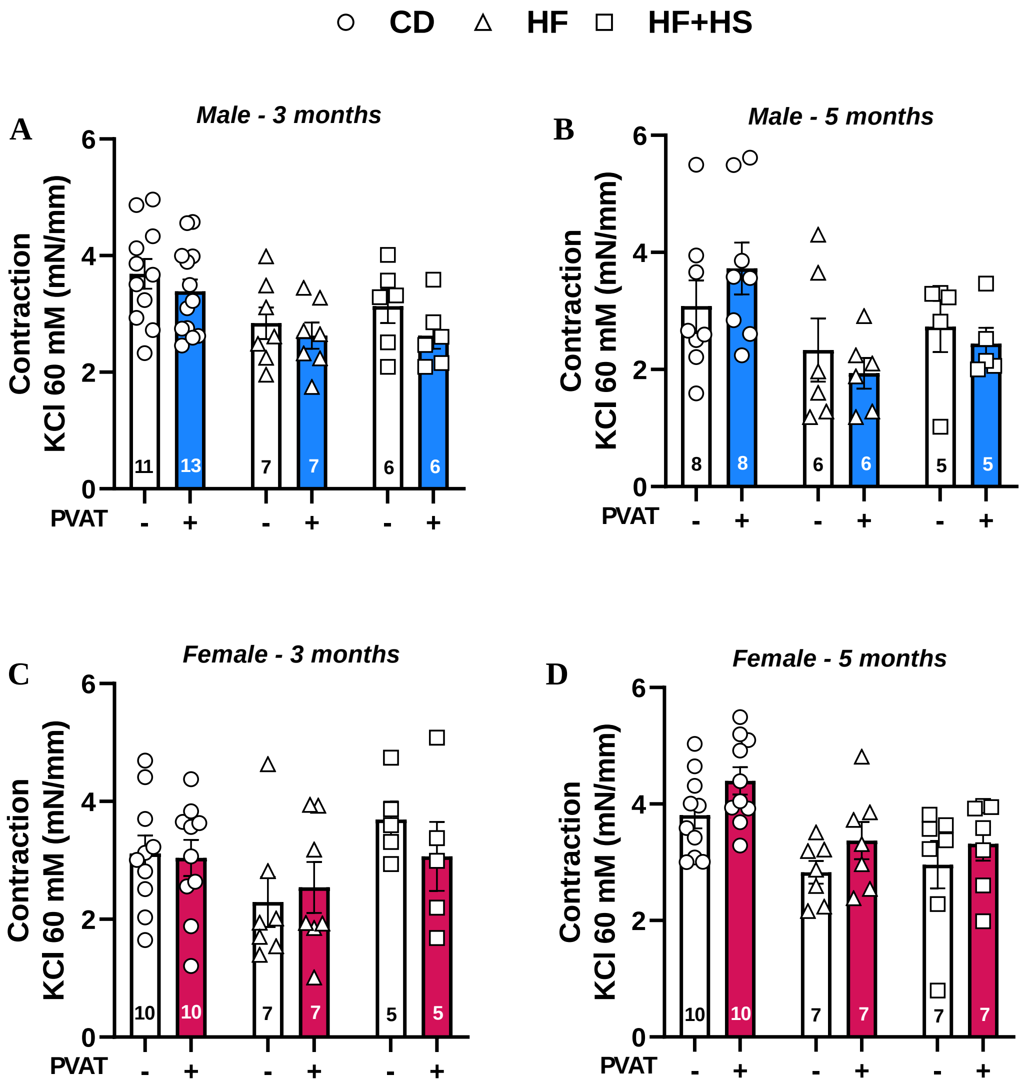

Figure S1. KCl-induced (60 mM) maximum contraction in mesenteric arteries without (-; open bars) or with (+; colored bars – male: blue and female: pink) perivascular adipose tissue (PVAT) of male (**A, B**) and female (**C, D**) mice fed with chow diet (CD, circle symbols), high-fat (HF; triangle symbols) or high-fat with high-sucrose (HF+HS; square symbols) diet for 3 (**A, C**) or 5 (**B, D**) months. Data are expressed as mean ± SEM; contraction is delta of maximum contractile response minus basal (mN/mm). The experimental number is included into the bars. Mann-Whitney test comparing PVAT- and PVAT+ in each group: $p>0.05$.

Table S3. Maximum response (Rmax) values (mN/mm) of phenylephrine-induced contraction in mesenteric arteries without (-) or with (+) PVAT of male and female mice fed with chow diet (CD), high-fat (HF) or high-fat with high-sucrose (HF+HS) diet for 3 or 5 months (mo).

Groups		Male			Female		
		CD	HF	HF+HS	CD	HF	HF+HS
3 mo	PVAT-	4.6±0.2 (7)	3.1±0.2 (6)	3.9±0.3 (6)	4.2±0.4 (11)	3.0±0.4 (7)	4.1±02 (5)
	PVAT+	4.3±0.3 (6)	2.5±0.4 (6)	2.5±0.1 (6)$^{\#}$	2.9±0.4 (8)	3.0±0.4 (6)	2.6±0.4 (4)
5 mo	PVAT-	3.9±0.4 (7)	4.3±0.4 (8)	3.7±0.5 (6)	4.7±0.2 (9)	3.5±0.2 (6)	3.3±0.5 (6)
	PVAT+	3.2±0.2 (5)	2.7±0.2 (6)$^{\#}$	1.8±0.2 (4)$^{\#}$	4.3±0.3 (10)	3.3±0.6 (6)	3.0±0.5 (5)

Data expressed as mean ± SEM. The experimental number used is into parenthesis. Mann- Whitney p>0.05 (5 mo male; 3 mo female). Two-way ANOVA: $^{\#}$p<0.05 *vs.* respective PVAT- group.
